# The endosome–lysosome pathway and information generation in the immune system^☆^

**DOI:** 10.1016/j.bbapap.2011.07.006

**Published:** 2012-01

**Authors:** Colin Watts

**Affiliations:** Division of Cell Signalling & Immunology, College of Life Sciences, University of Dundee, Dundee, DD1 5EH, UK

**Keywords:** Endosome–lysosome pathway

## Abstract

For a long time the lysosomal pathway was thought to be exclusively one for catabolism and recycling of material taken up by endocytosis from the external milieu or from the cytosol by autophagy. At least in the immune system it is clear now that endo/lysosomal proteolysis generates crucially important information, in particular peptides that bind class II MHC molecules to create ligands for survey by the diverse antigen receptors of the T lymphocyte system. This process of antigen processing and presentation is used to display not only foreign but also self peptides and therefore is important for ‘self’ tolerance as well as immunity to pathogens. Some cells, macrophages and particularly dendritic cells can load peptides on class I MHC molecules in the endosome system through the important, though still not fully characterised, pathway of cross-presentation. Here I try to provide a brief review of how this area developed focussing to some extent our own contributions to understanding the class II MHC pathway. I also mention briefly recent work of others showing that proteolysis along this pathway turns out to regulate immune signalling events in the innate immune system such as the activation of some members of the Toll-like receptor family. Finally, our recent work on the endo/lysosome targeted protease inhibitor cystatin F, suggests that auto-regulation of protease activity in some immune cells occurs. This article is part of a Special Issue entitled: Proteolysis 50 years after the discovery of lysosome.

## Highlights

► The original view of lysosomes as exclusively destructive organelles has changed. ► Controlled proteolysis in endo-lysosomes is needed for immunity. ► Antigen presentation and sensing of some microbial products requires protease action. ► Cystatin F may attenuate potentially toxic protease levels in some immune cells.

## 1 Introduction

In 1984 Christian de Duve published a monograph entitled: ‘A guided tour of the living cell’ [1]. It was based on a series of Christmas lectures he gave at Rockefeller University in the mid to late 70s to high school students. In the book, as no doubt in the lectures, he takes his young audience on a tour of the cell's diverse compartments encouraging them to imagine that they are ‘cytonauts’ exploring the cell's myriad internal structures. The book vividly describes the cell's major sub-compartments and what was then known about them. In his chapter on lysosomes he states that:

‘A few years ago, nobody in his senses would have dared to enter a cell by the endocytic route unless there were some strict guarantee that the lysosomal compartment would be bypassed…even if you escaped being burned by the acid or cut to pieces by the hydrolases, you would remain forever trapped within a membraneous prison’[1].

However, de Duve goes on to say that this view is changing and that there may be escape routes from the endo/lysosomal pathway. He does not give specific examples and not being an immunologist he probably was not thinking of experiments recently published in the then embryonic field of antigen presentation. In the early 1980s the lab of Emil Unanue, studying T cell responses to the pathogen *Listeria monocytogenes*, showed that before *Listeria monocytogenes* reactive T lymphocytes could bind to macrophages exposed to this bacterium a 30–60 min period was required during which the bacteria were partially catabolised following uptake into the macrophage phagolysosomal system [2]. The clear implication was that an intracellular processing event was needed before the T cells could recognised *Listeria* exposed macrophages and that some part of the bacterium was presumably being returned to the cell surface. Remarkably, T cells were still able to bind to the macrophages when they were fixed with aldehyde after the period of *Listeria* catabolism but not if the cells were fixed before exposure to the bacteria. Moreover, lysosomotropic agents such as chloroquine and ammonia, which reduced antigen catabolism, also reduced antigen presentation to T cells but again, not if applied after the period of *Listeria* uptake [3]. Unanue and colleagues speculated that this ‘processed antigen’ might be associated with the products of what were then known as I region genes and which we now identify as class II MHC molecules. This work followed pioneering studies from several labs which developed the concept of ‘MHC restriction’ in T cell responses to protein antigens: simply put, genes at the Major Histocompatibility Complex (MHC) locus controlled whether or not a particular inbred mouse strain responded to a protein or peptide antigen [4-6].

## 2 Early studies on antigen processing in the endo/lysosomal system

The work of Unanue's lab and other early studies indicated that complete destruction in the lysosomal system can indeed be avoided and that the material salvaged provides vitally important biological information. The next challenge was to determine what ‘antigen processing’ really entailed. Other model ‘antigens’, more tractable than *Listeria,* such as ovalbumin and hen egg lysozyme began to be used to move things forward. In a key study Grey and colleagues showed that aldehyde fixed antigen presenting cells (APC) could not present denatured ovalbumin to T cells but could present either chemically or enzymatically generated ovalbumin fragments [7]. These studies were among the first to show that partial antigen fragmentation was a necessary and sufficient event for T cell recognition and opened the way to defining the precise peptide sequences or ‘epitopes’ that T cells recognised. Soon after, experiments demonstrating direct binding of processed or synthetic antigenic peptides to isolated MHC class II molecules were performed demonstrating for the first time the likely biochemical basis of MHC restriction [8]. When the first crystal structure of a class I MHC molecule was solved in 1987 [9]and that for a class II MHC molecule a few years later [10], both showing clear evidence of electron density due to peptide in the groove between the two long α-helices, the endpoint of antigen processing was finally clear. In the intervening years many of the details of antigen processing leading to antigen presentation have been worked out, as of course has the molecular cell biology of membrane and protein traffic along the endocytic pathway.

This brief article is not an in depth review of this area which would require more space, more focus and less emphasis on our own work but rather, is a personal overview of some early and more recent developments that may be of interest to those from outside the field. Classical class I MHC peptide loading is not discussed since it takes place in the endoplasmic reticulum (ER) but so called cross-presentation on class I MHC molecules is mentioned since increasing evidence suggests that this non-canonical mode of peptide loading can take place in phagosomes or endosomes. At the end I mention some recent work from our lab which may suggest how de Duve's ferociously destructive lysosomal compartment might be tamed, particularly in those cells that elaborate toxic lysosomes to kill other cells or pathogens.

## 3 Antigen processing in B lymphocytes

The early studies outlined above demonstrated that antigen uptake was followed by a processing event and the association of proteolytically processed antigenic peptides with class II MHC molecules but many important questions were left open. In particular, where along the endocytic pathway did antigen processing take place? How was processed antigen transferred to class II MHC molecules and at what point in their biosynthetic itinerary? How much processing was needed and by which proteases? Were additional chaperones required and how similar were the events of antigen processing in macrophages and B cells? In the late 1980s when these questions came to the fore, dendritic cells were already known to be highly immunostimulatory for T cells [11] but were difficult to expand and work with *in vitro.* Consequently, early studies on the cell biology and biochemistry of antigen processing continued to be performed in B cells and macrophages.

It was well established that B cells required T cell ‘help’ to drive their differentiation into antibody secreting cells and eventually it became clear that, like macrophages, B cells captured antigen, processed it and reexpressed it linked somehow to MHC molecules. What was unclear was the role of the B cell antigen receptor (BCR), a membrane anchored form of antibody. Studies by Grey, Abbas [12, 13] and most definitively by Lanzavecchia [14] eventually showed that the BCR was a device for antigen capture for endo/lysosomal processing but was not involved in presenting antigen to T cells. In other words, recognition of antigen by B cells and T cells was sequential not simultaneous as earlier models had suggested. Human EBV-transformed B cells expressing BCRs specific for the tetanus toxin (TT) antigen were shown to present TT to human T cells at antigen concentrations as low as 10^− 12^ M, four orders of magnitude lower than that required by B cells that lacked a specific BCR for this antigen [14].

**Fig. 1:**
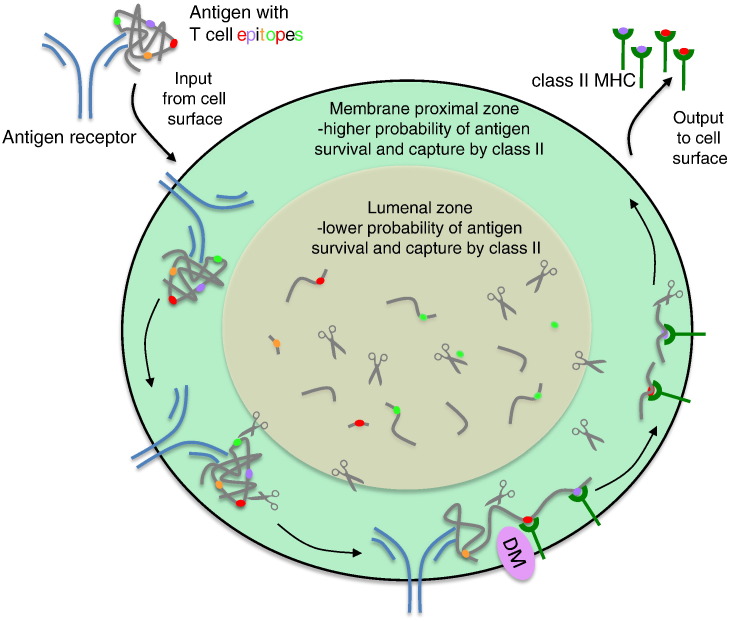
Antigen receptor directed antigen processing and ‘handover’ to class II MHC. The cartoon is meant to represent a class II MHC positive endo/lysosomal compartment in a B cell. BCR bound antigen (grey) with different potential T cell epitopes (coloured ovals) is delivered and processing commences whilst the antigen is still bound to the BCR. Scissors denote proteases. Limited cleavages produce large unfolded segments of antigen that can be captured by DM-chaperoned class II MHC molecules without release of antigen into the lumenal phase. Trimming (scissors) reduces the size of MHC associated antigen prior to delivery to the cell surface. NB vesicle trafficking steps not shown. The figure suggests that antigen fragments released into the lumen (orange) have a lower probability of survival and capture than fragments that are tethered by the antigen receptor and are membrane proximal (green). This scenario results in preferential presentation of some T cell epitopes over others. For experimental evidence see Ref. 18-20, 53.

I suspected that antigen-specific B cells could offer a valuable system to dissect the cell biology and biochemistry of antigen presentation since the fate of a single cohort of BCR-bound antigen molecules could be followed much as the endocytosis and subsequent fate of low density lipoprotein [15] or transferrin [16, 17] had been followed in the developing field of membrane and receptor trafficking. Using ^125^I-labelled TT we showed that this antigen was internalised through clathrin coated pits and that discrete fragments resolvable on SDS-gels were observed after about 20 min [18]. Antonio Lanzavecchia had provided us with several different EBV-transformed B cell clones specific for TT. Interestingly, the pattern of fragmentation was different in each clone and we showed that this was due to the fact that each had a different epitope specificity and that the substrate for processing was the TT/BCR complex and the BCR in each clone protected or ‘footprinted’ a distinct set of fragments [19]. Later we showed that this ‘steering’ of antigen processing by the BCR affected the parts of antigen displayed to T cells on class II MHC molecules[20]. Thus B cells with a defined epitope specificity presented some peptides, and could therefore collaborate with certain T cells, better than others. We also speculated at this time that the large antigen fragments attached to the BCR might be simultaneously engaged by local class II MHC molecules in the same membrane plane [18] but it took many years before we could show that this can indeed occur (Fig. 1 and see below). We obtained two other important results around this time in collaboration with Antonio Lanzavecchia. First, we showed using both biochemical and T cell assays that the lifetime of peptide/class II MHC complexes was in most cases equal to the lifetime of the MHC molecules themselves which in the case of EBV-transformed B cells was 25–35 h [21]. In other words, once formed, MHC/peptide complexes were very stable permitting antigen captured at a peripheral site *in vivo* to be presented in lymphoid tissue several days later. Second, using a direct biochemical assay for *de novo* formation of TT peptide/MHC complexes, we showed that processed antigen was captured by newly synthesised class MHC molecules prior to their arrival on the cell surface [22]. In other words, antigen capture was an integral part of the biosynthetic itinerary of class II MHC molecules and did not, for the most part, utilise a class II MHC recycling pathway we had recently demonstrated [23, 24]. This was in good agreement with work from others showing that class II MHC, but not class I MHC molecules, intersected the endocytic pathway on their way to the cell surface[25, 26] and that class II MHC molecules undergo a conformational change in a post-ER compartment upon invariant chain dissociation (see below) and peptide binding[27].

## 4 Mapping the events of antigen processing and class II MHC loading onto the endo/lysosomal pathway

An important issue, still not fully resolved, concerns the domains of the endocytic pathway where antigen processing occurs, where class II MHC molecules capture peptide and how these complexes are transported to the cell surface. In 1994, we and the groups of Ploegh and Mellman identified and isolated distinct compartments of the endocytic pathway that hosted peptide loading on class II MHC molecules [28-30]. We used a strategy in these and later studies first described by Aijoka and Kaplan [31] which permits the ablation of selected compartments of the endocytic pathway by loading them with horseradish peroxidase (HRP). By confining HRP to specific domains, for example early and recycling endosomes by conjugation to transferrin, compartment-specific ablation following addition of peroxide and diaminobenzidine (DAB) can be achieved. The dense cross-linked precipitate of DAB that forms effectively inactivates the HRP-containing compartment but remarkably, cellular traffic pathways that do not involve this compartment proceed normally, at least for a time. Using this approach we and the group of Sebastian Amigorena showed that to get to the site of Ii processing and peptide loading, class II MHC/Ii complexes must pass through transferrin receptor positive endosomes but that peptide loading itself occurs in later compartments [32, 33]. We further showed that, once formed, the itinerary taken by a specific assembled peptide/class II MHC complex to the cell surface did not overlap with itinerary of the recycling transferrin receptor since ablation of this domain did not prevent peptide/MHC expression on the cell surface [34]. It was also shown by Mellman, Amigorena and their colleagues that slowing the rate of invariant chain processing (see below) drove newly made class II MHC molecules deeper into the endocytic pathway suggesting that the differences that had been reported regarding the types of endosomes hosting class II MHC molecules might be explained by variations among cell types in the rate of invariant chain processing [35].

## 5 Removal of the invariant chain and peptide loading

While we were focussed on antigen processing, others were studying the biochemistry, genetics and structure of MHC proteins. Particularly relevant were studies on the biosynthesis and surface expression of class II MHC molecules. Several labs had shown that class II MHC α and β chains associated intracellularly with a third sub-unit named the invariant chain (Ii) but it was the finding of Blum and Cresswell that proteolysis is required to remove Ii from its partner MHC sub-units that allowed them to speculate that shedding of Ii, and capture of processed antigen might take place in the same compartment [36]. Peter Cresswell's lab went on to make the crucial finding that the association with Ii prevented binding of antigenic peptides to class II MHC molecules [37] and they further showed that some B lymphoblastoid cell lines that were defective in antigen presentation, accumulated Ii fragments on their class II MHC molecules [38]. These mutants isolated by Pious and others turned out to be in an MHC-like protein called DM, in human cells, HLA-DM [39]. Later work from several labs showed that human and murine DM catalyses the removal of a residual fragment of Ii generated following proteolytic processing and also stabilises the now empty peptide binding groove [40-42]. Moreover, sub-optimal peptides other than the Ii remnant can also be removed by the action of DM such that it acts as a peptide editor, driving the formation of the long-lived peptide/class II MHC complexes mentioned earlier [43]. The functions of a second class II MHC-like dimer HLA-DO have been more difficult to pin down but most labs agree that DO has an inhibitory effect on DM that may be relieved under particular conditions, for example in the compartments in B cells to which BCR bound antigen is delivered [44]. Inhibition of DM by DO is predicted to broaden the range of peptides presented facilitating tolerance to a greater variety of ‘self’ proteins. Consistent with this, Lisa Denzin's lab has recently shown that diabetes susceptible NOD mice became resistant to the disease when DO was overexpressed in dendritic cells [45].

## 6 Antigen processing

Advances in mass spectrometry in the early 1990s allowed several labs to analyse the peptides eluted from purified class II MHC molecules to reveal the peptide output of antigen processing in the endo/lysosomal pathway. These peptides were not strictly speaking from exogenous antigen but rather were derived from cellular and serum proteins that had access to the endocytic pathway. They were found to be longer and more variable in length than those bound to class I MHC and frequently featured nested sets, *i.e.* where a common core sequence was extended N- and C-terminally to varying degrees [46, 47]. Whereas the N and C termini of 8–10 residue class I MHC peptides form a network of hydrogen bonds with class I MHC residues located at the ends of the peptide binding groove [48] these interactions are absent in class II MHC molecules and instead the 12–19 (or longer) residue peptides eluted from purified class II MHC molecules protrude beyond the ends of the groove [48]. Our early biochemical studies on TT processing suggested that even longer antigen fragments were captured by class II MHC during physiological processing [22] and others showed that even unfolding of native antigen could be sufficient to form a complex, with class II MHC, stimulatory for a CD4 T cell [49]. What then does this say about the role of proteases in the class II MHC pathway? The main proteases found in the endo/lysosomal pathway are the cathepsins, some of which are cysteine proteases (cathepsins S,L,B,C,H and others) and some aspartyl proteases (cathepsins D & E) [50]. Together with the lab of Alan Barrett, we described a novel lysosomal cysteine protease with strict specificity for cleavage after asparagine residues [51, 52]. This asparagine endopeptidase (AEP) is homologous to the plant vacuolar enzyme legumain and is more closely related to the caspases than to cathepsins. AEP was discovered in antigen presenting cells because it dominated the processing of a 47kD C-terminal domain of the TT antigen *in vitro* (tetanus toxin C fragment or TTCF) when lysosomes from EBV-transformed B cells were used as a source of proteases. AEP makes only a few cleavages in TTCF at clearly identifiable Asn residues yet this is sufficient to generate antigen that can bind class II MHC molecules — further evidence that class II MHC can capture large antigen fragments [53]. In antigen presentation experiments conducted *in vitro*, mutagenesis of these Asn residues in TTCF or genetic ablation or chemical inhibition of AEP substantially reduced the efficiency of presentation of TTCF [54, 55]. An additional endo/lysosomal processing enzyme, though not a protease, has been shown to be important for presentation of some, though not all, T cell epitopes in antigens with internal disulphide bonds. The enzyme is γ-interferon induced lysosomal thiol reductase, or GILT and it catalyses the reduction of disulphide bonds likely rendering antigens containing them more susceptible to proteolytic processing [56]. Interestingly, Cresswell's lab, who discovered the role of GILT in class II MHC antigen processing, have recently shown that it can also be crucial, again for a sub-set of T cell epitopes, for viral antigens that are ‘cross-presented’ (see below) on class I MHC molecules [57].

As noted above, in B lymphocytes with high-affinity BCRs, antigen is processed as a complex with the receptor meaning that some antigen fragments will be tethered to the membrane surface [19]. We recently showed, in a system that used the cell surface as a surrogate antigen processing ‘compartment’, that large AEP-generated fragments of TTCF could be ‘handed over’ to adjacent class II MHC molecules in the membrane plane, *i.e.* without release into the bulk phase [53] much as we suggested many years ago [18]. Whether this ‘handover’ occurs during antigen processing in B cell class II MHC positive endosomes remains to be seen but confining the processing and MHC loading reaction to the 2-dimensional membrane plane has several appealing features (Fig. 1). It likely to result in faster antigen capture since diffusion into the lumen of the compartment is avoided. Consequently, it is likely to limit destructive antigen processing and competition from peptides free in the lumen of the loading compartment (Fig. 1). It may also explain the preferred relationships between B and T cell epitopes in protein antigens mentioned earlier. The late Eli Sercarz was much concerned with issues surrounding the processing and capture of T cell epitopes in protein antigens and, along with others promoted a ‘bind first trim later’ model for antigen capture by class II MHC, in part to explain his own data on immunodominance and ‘crypticity’ in protein antigens [58]. In this model MHC molecules compete with each other for binding to extended fragments of processed antigen which, once captured, are then trimmed by further processing. Our recent studies on TTCF strongly support the ‘bind first trim later’ model [53].

Our results on AEP and TTCF processing and presentation *in vitro* allowed us to propose that a small number of ‘unlocking’ cleavages by a single protease might generally suffice to generate a substrate for class II MHC capture [59]. That model is probably true in most cases. However, this is not to say that even very clear cut relationships between antigens and processing enzymes discovered *in vitro*, such as that between TTCF and AEP, signal an obligatory protease requirement *in vivo*, *i.e.* in immunised mice. We found recently that AEP deficient mice still raise effective immune responses to TTCF although they do so somewhat more slowly [60]. Two factors can explain why the *in vitro versus in vivo* requirements for AEP in TTCF presentation are different. First, the levels of AEP in antigen presenting cells *in vivo* are very low compared with B cell lines used *in vitro* so loss of AEP has a smaller impact. Second, the longer time scale of T cell activation and development of an antibody response *in vivo* offsets slower antigen processing by less optimal proteases. In other words, antigen presentation can ‘catch up’ blunting clear cut differences observed *in vitro*. Overall the antigen processing requirements in the class II MHC pathway appear to be quite minimal and quite redundant and a clear instance of an absolute requirement for a specific protease remains to be demonstrated. However, rather few antigens have been studied in protease deficient mice and other processing events in the endo/lysosome system do show non-redundant requirements for single enzymes *in vivo*. For example, AEP appears to be absolutely required to convert the single chain forms of cathepsins L, B and H to the two-chain forms that are found in the endo/lysosomal pathway [61].

## 7 Destructive antigen processing

Other antigens are preferentially cleaved by proteases other than AEP but the idea that a single protease making a few cleavages is sufficient to generate antigen stimulatory for T cells may generally apply. For example, in the case of the model antigen myoglobin processing by the aspartyl protease cathepsin D dominates when purified lysosomes are used as source of proteases and is sufficient to release T cell epitopes [62]. To our surprise however, dendritic cells lacking cathepsin D presented 2 different T cell epitopes in myoglobin better, not worse, compared with wild type cells. We showed that the requirement for aspartyl protease activity in cathepsin D null cells was satisfied by the presence of a related but less abundant aspartyl protease, cathepsin E [62]. Wild type cells simply contained too much aspartyl protease activity for optimal processing. In related studies Ira Mellman's lab showed that protein antigens that were resistant to processing were better immunogens compared with more easily processed antigens [63]. Taken together, the data suggest, rather counter-intuitively, that some antigens/vaccines might be made more immunogenic, or could be given in smaller doses, if they are harder to process. That could be achieved either by site directed mutagenesis to remove some processing sites or possibly by mixing the vaccine protein with protease inhibitors. Destructive processing might also compromise tolerance to ‘self’ proteins which must be presented either in the thymus or under particular conditions in other lymphoid organs to eliminate or inactivate autoreactive T cells. For example we found, together with David Wraith and colleagues, that AEP makes a cleavage in the middle of a well characterised ‘self’ epitope in myelin basic protein (MBP) that has been linked to the pathogenic T cell response in multiple sclerosis [64]. Whether or not this AEP cleavage compromises the induction of tolerance to this epitope in humans is not clear yet.

## 8 Cross-presentation and ER incorporation into phagosomes and endosomes

Class I MHC molecules acquire peptides not in the endo/lysosome pathway but in the endoplasmic reticulum (ER) following import of proteasome generated peptides through the TAP transporter system (reviewed in ref [65]). In the canonical class I MHC pathway these peptides are generated from newly synthesised proteins, for example, viral proteins. At first the concept that class I and class II MHC molecules presented peptides from the cytosol/nuclear compartment and from the endo/lysosomal compartments respectively was thought to be quite rigid. However, it became clear that while most cells indeed failed to present exogenous antigens on their class I MHC molecules, some could, particularly macrophages and dendritic cells [66]. This finding helped to explain early experiments by Bevan who demonstrated ‘cross-priming’ *in vivo* [67] and second, it could explain how CD8 T cell responses could be raised to viruses which do not infect dendritic cells. In other words, given the key role of dendritic cells in initiating CD8 immune responses to viruses, if class I MHC could only ever be loaded with peptides made biosynthetically, how would a response be made to a virus that does not infect DC [68]? By allowing some professional APC to load their class I MHC molecules with exogenous protein, virally infected cells, including necrotic or apoptotic cells, could be taken up by phagocytosis and viral antigens translocated into the cytosol for entry into the proteasome and TAP-dependent class I MHC loading pathway. Such a pathway would also permit CD8 T cell responses to tumour antigens, which are also not expressed within DC.

We and the lab of Ken Rock provided the direct evidence that macrophages and DC can translocate exogenous proteins from macropinosomes and phagosomes into the cytosol [69, 70] but working out the precise details of cross-presentation has taken some time and other labs have made the key advances here. A key development and a very interesting and controversial one in terms of the cell biology of the endocytic pathway, has centred on the idea that elements of the ER are incorporated into phagosomes and endosomes [71-73]. In other words instead of delivering exogenous antigen to the canonical site of class I MHC loading, the loading machinery, including class I MHC and TAP transporters is delivered to the antigen. To explain the proteasome-dependency of most cross-presentation, phagocytosed or endocytosed antigen is proposed to enter the cytosol transiently for processing and then to be re-imported into the mosaic ER-phago/endosome compartment [73, 72]. The controversy has mostly centred around whether or not the ER is a significant contributor to newly formed phagosomes and endosomes in dendritic cells and macrophages. Although some groups have not found much evidence for this [74] there is good functional evidence in living cells that both TAP and elements of the ER retro-translocation machinery are incorporated into and function in cross-presentation in dendritic cell endosomes and phagosomes [75, 76]. Thus in the current view, the ER retrotranslocation channel Sec61 and the ATPase p97 is used to shuttle antigens out of mosaic ER/phago/endosomes and TAP is used to shuttle proteasome-generated peptides back in.

It should also be mentioned that a distinct pathway of cross-presentation was also described which does not require proteasome activity or TAP transporters [77]. In this pathway processing within phagosomes or endosomes generates suitable peptides for loading onto class I MHC molecules present in those compartments. More recent work identified a role for cathepsin S in this pathway [78]. There are many other interesting aspects of cross-presentation that cannot be covered here including its greater prominence in certain DC types and preferential access to the ‘cross-presenting’ compartment by certain surface receptors. For a recent review see [79].

## 9 Endosomal proteolysis and Toll-like receptor signalling

Toll-like receptors (TLRs) are expressed on many cells and signal the presence of so called pathogen associated molecular patterns found, for example, in bacterial cell wall material and viral nucleic acids. In macrophages and particularly DC, TLR signalling triggers a variety of responses which direct and enhance the performance of the adaptive immune system. For example, DC challenged with TLR ligands increase their rate of antigen uptake and processing, reorganise their cytoskeleton and vacuolar compartments and increase cell surface expression of MHC molecules and costimulatory molecules along with the chemokine receptor CCR7 which is required for DC migration to lymph nodes. This very active area has been extensively reviewed by ourselves and others [80, 81]. Here I mention one specific aspect relating to the theme of this volume: proteolysis. TLRs 3, 7, 8 and 9 detect specific configurations of RNA and DNA that are often more abundant in bacteria and viruses than in mammalian cells. For example, TLR9 recognises CpG motifs that are under-methylated (relative to mammalian DNA) and TLR3 recognises double-stranded RNA. Apparently to improve discrimination between pathogen and ‘self’ nucleic acids it turns out that these TLRs signal not from the plasma membrane but from endosomal compartments where viral and bacterial nucleic acid is most likely to be released. Interestingly, for TLR9 to signal efficiently it must first be proteolytically processed. Approximately half of the N-terminal ectodomain is removed upon entry into the endocytic pathway and importantly only this form of TLR9 can engage the key signalling adaptor MyD88 [82, 83]. Recent studies have begun to identify the proteases responsible: both AEP and the cathepsins are involved [84]. These are remarkable results: whereas signalling from growth factor receptors is often terminated by proteolysis in the endocytic pathway – the EGF receptor comes immediately to mind – proteolysis turns out to be a necessary precursor for signalling from TLR9 and probably the other nucleic acid sensing TLRs as well.

## 10 Cystatin F may be a ‘cytoprotectant’ in some immune cells

The events of antigen presentation and TLR signalling show that the hostile and destructive lysosomal environment so vividly described by Christian de Duve in ‘A Guided Tour of the Living Cell’ is in fact compatible with information generation and signal processing in the immune system. But how can the hydrolases found in these compartments be kept under control? Several mechanisms seem to operate, at least in DC. Many lysosomal proteases work optimally at acidic pH explaining in part the relatively benign environment found in ‘early’ endosomes and the increase in hydrolase activity as the endosome–lysosome pathway is traversed. Dendritic cells express lower levels of lysosomal proteases than macrophages reducing the likelihood of antigen destruction [85]. In addition they recruit the NADPH oxidase NOX2 to alkalinise newly formed phagosomes, further limiting the action of potentially destructive proteases and improving antigen cross-presentation [86]. A further intriguing attenuator of lysosomal proteolysis in APC is the p41 variant of the more abundant p31 form of the invariant chain mentioned earlier. The additional 64 amino acids found in p41 acts as an inhibitor of cathepsin L and other endo/lysosomal proteases [87, 88]. Since p41 is particularly abundant in DC, it may also limit destructive antigen processing, maximising the chance of successful presentation of processed antigen. Finally we have recently shown how an unusual member of the cystatin family of cysteine protease inhibitors, cystatin F, may also limit protease activity in the endo-lysosome pathway, not just in DC but in several immune cells types where it is selectively expressed.

**Fig. 2:**
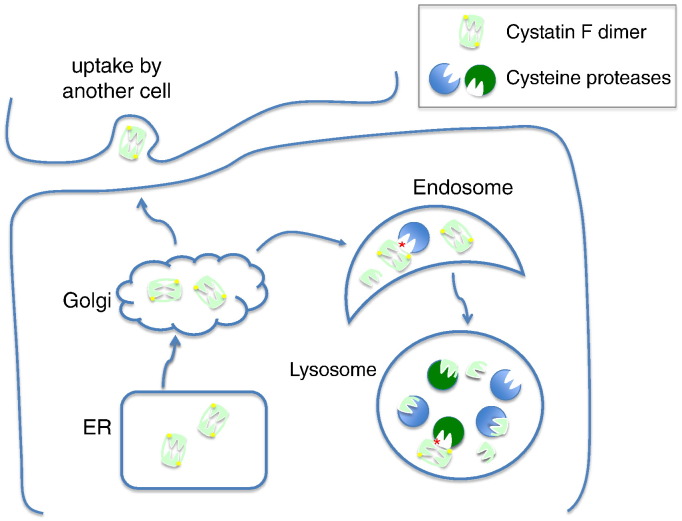
Cystatin F as an attenuator of lysosomal cysteine proteases. Cystatin F (pale green) dimerises in the ER through di-sulphide linkages (yellow dots) and is transported to the endocytic pathway using the mannose-6-phosphate targeting system. Dimer to monomer conversion is driven by proteolytic action (red asterisk) on an N-terminal linking peptide that contains one of the cysteines involved in the disulphide bridge. Protease generated monomer has an N-terminus truncated by 15 residues relative to monomer generated by reducing agents *in vitro*. Since cystatin N-termini are important in cysteine protease inhibition, this changes the target range of the monomer. For example, cathepsin C is blocked by protease generated monomer but not by full-length monomer generated by reduction. Secreted cystatin F can be taken up, again via mannose-6-phosphate receptors and activated by bystander cells. For experimental evidence see references 89-95.

The cystatins are a family of low molecular weight, tight binding inhibitors of cysteine proteases [89]. Some members are found in the cytosol but the majority are secreted and found in body fluids and tissue interstitia where they are proposed to ‘mop up’ inadvertently released proteases and prevent tissue damage. Cysatin F, also known as leukocystatin, does not conform to this model. Although it is made with a signal sequence, only a fraction is secreted, the rest being targeted to the endo-lysosome pathway [90] due to mannose-6-phosphate receptor recognition of its N-linked saccharides [91]. Importantly, this material and any that is secreted, is initially inactive. This is because it is made as a disulphide linked dimer [92] and partnering with another molecule of cystatin F precludes binding to proteases [93, 94]. Although reduction of the dimer can generate active cystatin F *in vitro*, high levels of reducing agent are needed [93] and we recently showed that in living cells inactive dimer to active monomer conversion is achieved by protease action on the extended N-terminal regions that link the partner subunits [95]. The discovery of a protease inhibitor that is itself activated by proteolysis immediately suggests that cystatin F may provide negative feedback regulation of excessive protease activity (Fig. 2). But under what specific circumstances might this be needed? The cell types that express cystatin F may offer an important clue. The inhibitor is expressed in cytotoxic T cells, NK cells, NKT cells, mast cells, neutrophils, eosinophils and some other cell types. All these cells elaborate granules, which are in effect lysosomes, that can be secreted in a regulated manner, to achieve their effector function, for example killing of target cells or pathogens [96]. The contents of these granules are toxic suggesting that strategies to limit their activity until required may be useful to prevent self-inflicted injury. Consistent with a role for cystatin F in such protection, we have found that one of the protease targets of cystatin F is cathepsin C [95], the cysteine protease that activates the granzymes in cytotoxic T cells and NK cells and several of the effector proteases of neutrophils (elastase and cathepsin G) and mast cells (chymase). We are currently studying the phenotype of cystatin F null mice to establish its *in vivo* role.

Finally some cystatin F is secreted and unlike monomeric cystatins, will not bind to and therefore be quenched, by proteases outside cells. Being glycosylated it can be readily taken up through carbohydrate recognition receptors such as the mannose-6 phosphate receptor and subsequently activated in the recipient cell [91]. Thus protease activity may be modulated *in trans* by cystatin F expressing cells (Fig. 2) a facility which may permit the attenuation of some of the less desirable phenomena that have been linked to the cysteine cathepsins such as inflammation and tumour progression.

## 11 Closing summary

Our understanding of the endo/lysosomal pathway has come a long way since the pioneering studies of de Duve and colleagues. Complete destruction of material taken into cells by endocytosis, is certainly not the only and may not even be the most important function of the endo/lysosome system in many cells. In immune cells such as DC crucially important information is generated that activates both the innate and adaptive immune systems. This information consists of protein fragments that complex with class II MHC molecules to create a ‘currency’ recognised by the CD4 T lymphocyte system. Class I MHC molecules can also be loaded in the endocytic pathway through the still to be fully characterised mechanisms of cross-presentation which intriguingly, seems to involve incorporation of limited amounts of ER into phagosomes and endosomes. Some pathogen sensing TLR receptors are activated by endosomal proteolysis. Finally, the toxic enzymes elaborated by various immune cells and packaged into lysosome like organelles for discharge onto target cells may be hazardous to the cell producing them. Cystatins and perhaps particularly cystatin F, which is itself activated by proteolysis, may act as a brake and protect cells from their own armoury of toxic enzymes.
